# *Rickettsia slovaca* in *Dermacentor marginatus* and Tick-borne Lymphadenopathy, Tuscany, Italy

**DOI:** 10.3201/eid1405.070976

**Published:** 2008-05

**Authors:** Marco Selmi, Luigi Bertolotti, Laura Tomassone, Alessandro Mannelli

**Affiliations:** * Osservatorio Permanente per Patologie a Trasmissione Vettoriale, Lucca, Italy; † Università degli Studi di Torino, Grugliasco, Italy; 1 Current affiliation: European Food Safety Authority, Parma, Italy

**Keywords:** Tick-borne lympadenopathy, Rickettsia slovaca, Dermacentor marginatus, surveillance system, dispatch

## Abstract

Of 263 patients in Tuscany, Italy, from whom ticks were removed during July 2005–May 2007, five showed signs of tick-borne encephalopathy. Of the ticks, 17 were *Dermacentor marginatus*; 6 (35.3%) of these were identified by sequence analysis as containing *Rickettsia slovaca*. Tick-borne lympadenopathy occurs in this area.

*Rickettsia slovaca* was first isolated in Czechoslovakia from the tick vector *Dermacentor marginatus* in 1968 (*1*) and was subsequently detected in several European countries. It was recognized as the causative agent of tick-borne lymphadenopathy (*2*–*4*) and *Dermacentor* spp.–borne necrosis-erythema-lymphadenopathy (*5*). Typical clinical signs of infection include skin lesions at the tick bite site and regional, often painful, lymphadenopathy (*2*,*3*). Acute disease can be followed by residual alopecia at the bite site (*2*). This disease is considered a mild rickettsiosis, but severe symptoms have been described, especially in untreated patients (*2*).

*D*. *marginatus* is the only member of the species *Dermacentor* reported in Italy; it is widely distributed in prairies and steppes up to 2,500 m above sea level, including the northern Apennines (*6*). Adults are active within a temperature range of 4°C to 16°C (*7*,*8*). Temperature influences the seasonality of tick-borne lymphadenopathy, which has a higher incidence during cold months (*4*,*9*). We describe results from a tick-borne zoonoses surveillance system that was implemented in 2002 at the Lucca local health unit (ASL 2) in Tuscany, Italy.

## The Study

Patients admitted to emergency units in Tuscany, Italy, for tick removal were followed up for 40 days. Epidemiologic and clinical data were collected for each patient by using a standardized questionnaire. History of allergic reactions or hypersensitivity to tick bites was considered and evaluated to avoid mistakes in case definition.

**Figure 1:**
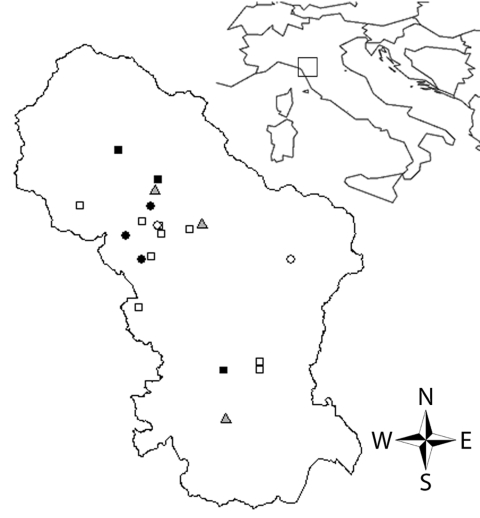
Distribution of tick-borne lymphadenopathy cases in Tuscany, Italy. Circles indicate cases, squares indicate patients bitten by *Dermacentor marginatus* who were not classified as case-patients, and triangles indicate emergency units. Negative (white symbols) and positive (dark symbols) PCR results for spotted fever group rickettsiae are indicated.

Ticks were classified by using standard identification keys (*8*) and stored in 70% ethanol until DNA extraction. *D*. *marginatus* females were measured, and the degree of engorgement (tick engorgement index [TEI]) was visually estimated. Ticks were ranked by 3 TEI levels: 1 = completely unengorged, 2 = intermediate (idiosoma length ≈2× scutum width), and 3 = engorged (idiosoma length >2× scutum width). Association between TEI levels and occurrence of clinical symptoms was evaluated by using the Fisher exact test. All statistical analyses were conducted by using R statistical software (*10*). Arcview 3.3 (Environmental Systems Research Institute Inc., Redlands, CA, USA) was used to map the geographic distribution of cases in the study area (Figure 1).

For pathogen detection by PCR, ticks were individually homogenized with a pestle in microcentrifuge tubes and DNA was extracted with the DNeasy Blood and Tissue Kit (QIAGEN, Hilden, Germany). Negative controls (distilled water) were used to check for contamination of samples during this phase. Success of DNA extraction was verified by using PCR for tick mitochondrial 16S rDNA (*11*). Two PCR assays, targeting citrate synthase A (*gtl*A) and outer membrane protein A (*omp*A) genes, were used to identify spotted fever group rickettsiae as described (*12*).

Cases of abnormal tickbite reaction were observed only in patients bitten by *D*. *marginatus* (Table 1). From July 2005 through May 2007, information on 263 patients was recorded in the surveillance system. Removed ticks were classified as *Ixodes ricinus* (n = 187), *Rhipicephalus sanguineus* (n = 6), or *D*. *marginatus* (n = 17); 53 were unclassified (lost or disrupted).

**Table 1:** Patients bitten by *Dermacentor mariginatus* and admitted to emergency units, Tuscany, Italy

Patient no.	Year of birth	Sex	Site of tick bite	TEI*	Symptoms	Therapy 1†	Therapy 2‡	PCR result§	Tick-borne lymphadenopathy
31	2005	F	Head	1	Small nodules		No		
60	1937	M	Trunk	2	Itching	Yes			
89	2003	M	Head	1	No		No	*Rickettsia slovaca*	
117	1943	F	Trunk	3	Wet painful rash (20 mm) over 12 mo	Yes			
121	1954	F	Arm	M	None		No		
138	1968	M	Head	1	None		No		
145	2001	F	Head	3	Enlarged cervical lymph node, painful lymph node, fever, alopecia		15	*R. slovaca*	Yes
149	1939	F	Head	2	Fever		10	*R. slovaca*	
154	1930	M	Trunk	1	None				
155	1968	F	Head	2	Enlarged cervical lymph node, painful lymph node, fever, weariness		15		Yes
159	1950	M	Trunk	M	Itching		No		
175	2000	M	Head	2	Enlarged cervical lymph node, tache noire, alopecia		15		Yes
252	1968	F	Head	1	Enlarged cervical lymph node, painful lymph node, weariness, myalgia		15	*R. slovaca*	Yes
254	1949	F	Head	3	None				
256	1949	F	Trunk	2	Tache noire, itching, small nodules		15	*R. slovaca*	
263	2002	F	Head	M	Fever, headache		28¶ + 15#		
266	2001	M	Head	3	Pain at tick bite site, enlarged, painful cervical lymph node		21¶ + 12#	*R. slovaca*	Yes

*TEI, tick engorgement index based on visual evaluation for female ticks: 1, completely unengorged; 2, intermediate (idiosoma length ≈2× scutum width); 3, engorged (idiosoma length >2× scutum width). M, male tick. †Therapy administered (doxycycline) when patients were discharged from emergency unit. ‡Therapy administered (doxycycline) when disease was diagnosed; numbers indicate duration of treatment in days. §Pathogen identification by outer membrane protein A gene sequencing. ¶Amoxicillin. #Clarithromycin.

**Figure 2:**
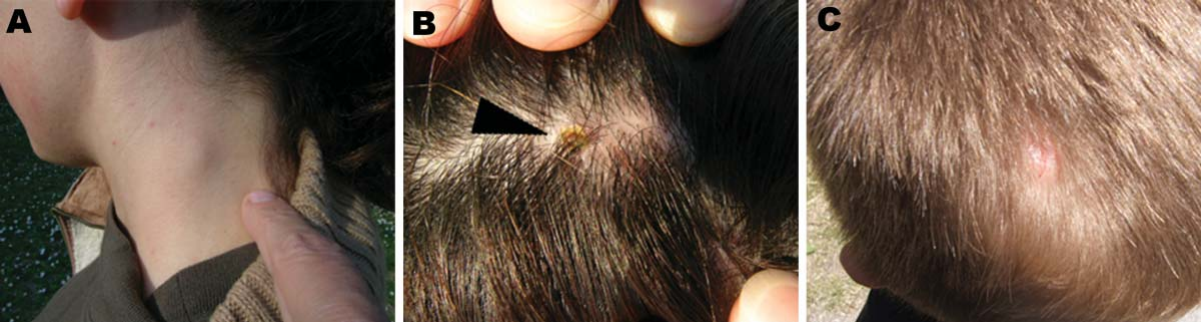
Enlarged lymph nodes (A), tache noire (arrowhead) (B), and alopecia (C) in patients admitted to the Lucca local health unit, Tuscany, Italy.

Five patients (29.4%) (2 adults and 3 children) showed clinical signs typically related to tick-borne lymphadenopathy. We defined tick-borne lymphadenopathy case-patients as those with skin lesion (eschar) at the tick bite site and regional lymphadenopathy (*2*). The 5 patients were examined by physicians at least twice. Each patient had enlarged lymph nodes (Figure 2 , panel A) at the second examination. Crusted scalp lesions (Figure 2, panel B) ranged in diameter from 8 mm to 25 mm. One patient was surgically treated to remove necrotic tissue from a large tache noire; he showed alopecia >8 months after acute episode (Figure 2, panel C). Three cases were recorded in spring, 1 in autumn, and 1 in winter (Table 2). Association between *Dermacentor* TEIs and occurrence of symptoms was not statistically significant (p = 0.13). Six (35.3%) of 17 ticks were positive by PCR for either *gtl*A or *omp*A. The *ompA* gene sequences of all positive samples showed similarity of 100% with the *R*. *slovaca* (GenBank accession no. U43808).

**Table 2:** Temporal distribution of *Dermacentor* spp. bites and tick-borne lymphadenopathy cases, Tuscany, Italy, 2006

Characteristic	Jan	Feb	Mar	Apr	May	Jun	Jul	Aug	Sep	Oct	Nov	Dec
*Dermacentor* spp. tick bite	0	0	0	4	2	0	3	0	1	4	1	2
Tick-borne lymphadenopathy cases	0	0	0	2	1	0	0	0	0	1	0	1

## Conclusions

Until recently, Mediterranean spotted fever (MSF) caused by *R*. *conorii* and transmitted by the brown dog tick, *R. sanguineus*, was considered the only tick-borne rickettsiosis in Italy. Local investigations at ASL 2 showed a decrease in MSF incidence during the past 10 years and fewer *Rhipicephalus* spp. bites than *Ixodes* spp. and *Dermacentor* spp. bites. In the past 2 years, no MSF was officially recorded at ASL 2, and our results suggest an emerging role of *R*. *slovaca* as a tick-borne pathogen in the area.

Most bitten patients showed specific clinical manifestations (fever, itching, rash, weariness, and myalgia) and 5 (29.4%) had typical signs of tick-borne lymphadenopathy. Pathogen identification in ticks agreed with the case definition in 50% of cases. Infected ticks were removed from 3 patients not considered to have lymphadenopathy; 1 patient showed no symptoms, probably because the tick was not attached long enough to enable pathogen transmission (TEI = 1).

A study reported that children and women have a higher risk than men for infection caused by *R*. *slovaca* (*4*); all of our patients with tick-borne lymphadenopathy were children or women. This result reflects the higher risk for bite by *Dermacentor* spp.; 6 (35.3%) of the 17 patients were <10 years of age and 10 (58.8%) were female.

Raoult et al. reported that all cases of tick-borne lymphadenopathy cases recorded in their study were found from October through May (*4*), confirming the seasonal incidence of tick-borne lymphadenopathy in cold months (*2*). The activity of *D. marginatus* adults in cold months has been reported (*7*,*8*). Our results confirm this trend, although the absence of cases of tick-borne lymphadenopathy in winter can be explained by the lower human frequency of high-risk areas (Table 2).

Cases reported in this study were concentrated in the northern part of ASL 2, despite the lower population density (Figure 1). Local investigations showed that wild boars, which seem to be important in the epidemiology of *R*. *slovaca* (*13*), are found in this area. In the northern part of this area, woodland habitat and human habits increase the risk for human contact with vectors. TEI did not show any association with cases but study of feeding duration is so far strictly applied to the transmission of *Borrelia burgdorferi* s.l. by *I. ricinus* (*14*).

In recent years, many efforts have been made to characterize distinct tick-borne diseases. A diagnostic approach that includes surveillance of patient symptoms and vectors can be helpful in identifying cases of tick-borne lymphadenopathy (*15*). Tick identification is also important in diagnosis (*4*).

Our data indicate a high prevalence of *R*. *slovaca* in *D*. *marginatus* collected from patients. On the basis of these results, all patients bitten by *D*. *marginatus* should be observed to determine whether specific treatments are required. The new surveillance system in Lucca will provide real-time data that will be useful for evaluation of patient health problems.

## References

[1] Rehacek J. *Rickettsia slovaca*, the organism and its ecology. Acta Scientifica National Academy Scientifica Bohemoslovacae Brno. 1984;18:1–50.

[2] Lakos A. Tick-borne lymphadenopathy (TIBOLA). Wien Klin Wochenschr. 2002;114:648–54. 12422619

[3] Lakos A. Tick-borne lymphadenopathy—a new rickettsial disease? Lancet. 1997;350:1006. 10.1016/S0140-6736(05)64072-X 9329524

[4] Raoult D, Lakos A, Fenollar F, Beytout J, Brouqui P, Fournier PE. Spotless rickettsiosis caused by *Rickettsia slovaca* and associated with *Dermacentor* ticks. Clin Infect Dis. 2002;34:1331–6. 10.1086/340100 11981728

[5] Oteo JA, Ibarra V, Blanco JR, Martinez de Artola V, Marquez FJ, Portillo A, *Dermacentor*-borne necrosis erythema and lymphadenopathy: clinical and epidemiological features of a new tick-borne disease. Clin Microbiol Infect. 2004;10:327–31. 10.1111/j.1198-743X.2004.00782.x 15059122

[6] Mannelli A, Tolari F, Pedri P, Stefanelli S. Spatial distribution and seasonality of ticks (Acarina: Ixodidae) in a protected area in the northern Apennines. Parassitologia. 1997;39:41–5. 9419846

[7] Rukhkian MI. Vertical migrations of hungry imagoes of the tick *Dermacentor marginatus* [in Russian]. Parazitologiia. 1987;21:680–3. 2963257

[8] Manilla G. Acari, Ixodida (fauna d’Italia 36): Bologna (Italy): Edizioni Calderoni; 1998.

[9] Raoult D, Weiller PJ, Chagnon A, Chaudet H, Gallais H, Casanova P. Mediterranean spotted fever: clinical, laboratory and epidemiological features of 199 cases. Am J Trop Med Hyg. 1986;35:845–50. 3728799 10.4269/ajtmh.1986.35.845

[10] R Development Core Team. R: a language and environment for statistical computing. 2007 [cited 2008 Jan 31]. Available from http://www.R-project.org

[11] d’Oliveira C, van der Weide M, Jacquiet P, Jongejan F. Detection of *Theileria annulata* by the PCR in ticks (Acari:Ixodidae) collected from cattle in Mauritania. Exp Appl Acarol. 1997;21:279–91. 10.1023/A:1018455223462 9203350

[12] Bertolotti L, Tomassone L, Tramuta C, Grego E, Amore G, Ambrogi C, *Borrelia lusitaniae* and spotted fever group rickettsiae in *Ixodes ricinus* (Acari: Ixodidae) in Tuscany, central Italy. J Med Entomol. 2006;43:159–65. 10.1603/0022-2585(2006)043[0159:BLASFG]2.0.CO;2 16619594

[13] Ortuno A, Quesada M, Lopez-Claessens S, Castella J, Sanfeliu I, Anton E, The role of wild boar (*Sus scrofa*) in the eco-epidemiology of *R. slovaca* in northeastern Spain. Vector Borne Zoonotic Dis. 2007;7:59–64. 10.1089/vbz.2006.0576 17417958

[14] Gray J, Stanek G, Kundi M, Kocianova E. Dimensions of engorging *Ixodes ricinus* as a measure of feeding duration. Int J Med Microbiol. 2005;295:567–72. 10.1016/j.ijmm.2005.05.008 16325552

[15] Brouqui P, Bacellar F, Baranton G, Birtles RJ, Bjoersdorff A, Blanco JR, Guidelines for the diagnosis of tick-borne bacterial diseases in Europe. Clin Microbiol Infect. 2004;10:1108–32. 10.1111/j.1469-0691.2004.01019.x 15606643

